# Post-prandial muscle protein synthesis rates following the ingestion of pea-derived protein do not differ from ingesting an equivalent amount of milk-derived protein in healthy, young males

**DOI:** 10.1007/s00394-023-03295-6

**Published:** 2024-01-16

**Authors:** Philippe J. M. Pinckaers, Joey S. J. Smeets, Imre W. K. Kouw, Joy P. B. Goessens, Annemarie P. B. Gijsen, Lisette C. P. G. M. de Groot, Lex. B. Verdijk, Luc J. C. van Loon, Tim Snijders

**Affiliations:** 1 TiFN, Wageningen, The Netherlands; 2 Department of Human Biology, NUTRIM School of Nutrition and Translational Research in Metabolism, Maastricht University Medical Centre+, Maastricht, The Netherlands; 3 Division of Human Nutrition and Health, Department of Agrotechnology and Food Sciences, Wageningen University, Wageningen, The Netherlands

**Keywords:** Plant-based proteins, Dairy, Fractional synthesis rate, Amino acids

## Abstract

**Purpose:**

Plant-derived proteins have received considerable attention as an alternative to animal-derived proteins. However, plant-derived proteins are considered to have less anabolic properties when compared with animal-derived proteins. The lower muscle protein synthesis rates following ingestion of plant- compared with animal-derived protein have been attributed to the lower essential amino acid content of plant-derived proteins and/or their specific amino acid deficiencies. This study aimed to compare post-prandial muscle protein synthesis rates following the ingestion of 30 g pea-derived protein with 30 g milk-derived protein in healthy, young males.

**Methods:**

In a randomized, double-blind, parallel-group design, 24 young males (24 ± 3 y) received a primed continuous L-[ring-^13^C_6_]-phenylalanine infusion after which they ingested 30 g pea (PEA) or 30 g milk-derived protein (MILK). Blood and muscle biopsies were collected frequently for 5 h to assess post-prandial plasma amino acid profiles and subsequent post-prandial muscle protein synthesis rates.

**Results:**

MILK increased plasma essential amino acid concentrations more than PEA over the 5 h post-prandial period (incremental area under curve 151 ± 31 *vs* 102 ± 15 mmol∙300 min∙L^−1^, respectively; *P* < 0.001). Ingestion of both MILK and PEA showed a robust muscle protein synthetic response with no significant differences between treatments (0.053 ± 0.013 and 0.053 ± 0.017%∙h^−1^, respectively; *P* = 0.96).

**Conclusion:**

Post-prandial muscle protein synthesis rates following the ingestion of 30 g pea-derived protein do not differ from the response following ingestion of an equivalent amount of milk-derived protein. International Clinical Trials Registry Platform (NTR6548; 27–06-2017).

**Supplementary Information:**

The online version contains supplementary material available at 10.1007/s00394-023-03295-6.

## Introduction

Protein ingestion increases muscle protein synthesis rates [1, 2]. The increase in muscle protein synthesis rate is driven by the post-prandial increase in plasma essential amino acid (EAA) concentrations [3], with the rise in circulating leucine concentration being of particular relevance [4–8]. The anabolic properties of different proteins or protein sources seem to be largely determined by their EAA content, amino acid profile, and their protein digestion and amino acid absorption kinetics [9–11]. Consequently, post-prandial muscle protein synthesis rates can differ substantially following ingestion of the same amount of protein derived from different protein sources [12–14].

Within the wide variety of dietary protein sources, the main categories are animal (e.g., milk and meat) and non-animal proteins (e.g., wheat and soy). Within the non-animal proteins, plant proteins comprise a large part of our daily protein intake [15] and are likely to become more important with respect to future global protein needs and more sustainable protein production [16, 17]. However, plant-derived proteins are considered to have lesser anabolic properties when compared to animal-derived proteins, due to their lower digestibility and incomplete amino acid profile [17, 18]. So far, only a few studies have directly compared the muscle protein synthetic response following the ingestion of a plant-derived protein versus high(er) quality animal-derived proteins, demonstrating equivocal results, with muscle protein synthesis rates being either lower [14, 19–21], higher [14], or not different [22–24]. Furthermore, these studies have mainly focused on investigating soy- [14, 20–22] and wheat- [19, 25] derived proteins (and more recently also potato-derived protein [24]). Most plant-derived proteins are generally low in essential amino acid content and often deficient in one or more specific amino acids, particularly leucine, lysine, and/or methionine [26]. The amino acid composition and deficiencies can be quite variable between different plant-based proteins. To what degree this may have an impact on their properties to stimulate post-prandial muscle protein synthesis rates remains to be determined.

Pea-derived protein has received considerable interest as an alternative for animal-derived proteins, as together with soy protein it forms one of the main plant-based protein sources used in meat substitutes [27–30]. Pea-derived protein is considered of interest given its high nutritional value, availability, non-allergenic properties, and low production costs [31]. Pea-derived protein contains a sufficient amount of total essential amino acids (30%) and has a leucine (7.2%) and lysine content (5.9%) that exceeds the WHO/FAO/UNU amino acid requirements [32]. The latter is the proposed amino acid requirement that indicates the amount of amino acids that needs to be ingested to maintain skeletal muscle mass in healthy adults [32]. However, total essential amino acid content of pea-derived protein is less when compared with most animal-based proteins. Furthermore, pea-derived protein is particularly low in methionine. Whether this lower total essential amino acid content and low methionine content compromises the capacity to stimulate post-prandial muscle protein synthesis remains to be assessed.

In the present study, we aimed to compare the impact of ingesting 30 g pea- *vs* 30 g milk-derived protein on post-prandial muscle protein synthesis rates in vivo in healthy, young males. We hypothesize that the ingestion of 30 g pea-derived protein would result in lower post-prandial muscle protein synthesis rates when compared with the ingestion of an equivalent amount of milk-derived protein.

## Subjects and methods

### Participants

A total of 24, healthy, recreationally active males aged 18–35 years were recruited to participate in this parallel-group, double-blind, randomized controlled trial to compare the impact of ingesting 30 g pea and 30 g milk-derived protein on post-prandial muscle protein synthesis rates in vivo in humans. As we provided the same absolute amount of protein (30 g), we decided to select only a single sex in the present study, to limit the range of the amount of protein provided when expressed per kg muscle mass. Participants were recreationally active and generally performed between 2 and 4 exercise sessions per week in various sports (e.g., soccer, basketball, weight lifting, running, and cycling), but were not involved in any structured progressive exercise training regimen. This study was part of a larger trial registered at the International Clinical Trials Registry Platform (NTR6548) and was conducted between June 2017 and April 2019 at Maastricht University in Maastricht, The Netherlands (see Supplemental Fig. 1 for the CONSORT (Consolidated Standards of Reporting Trials) flow diagram, indicating the specific comparison that the current study was based on). The data of the milk-derived protein group were used in various comparisons and, as such, have been published previously, as well as the procedures applied in this trial [23, 33]. All participants were informed about the purpose of the study, the experimental procedures, and possible risks before providing informed written consent to participate. The procedures followed were in accordance with the ethical standards of the medical ethics committee of Maastricht University Medical Centre + (METC 173001), and in accordance with the Helsinki Declaration of 1975 as revised in October 2013. The study was independently monitored and audited by the Clinical Trial Centre Maastricht.

**Fig. 1:**
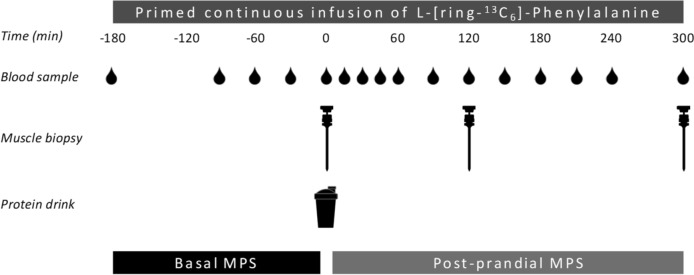
Schematic representation of the experimental design

### Preliminary testing

Participants aged 18–35 years, with BMI > 18.5 and < 27.5 kg∙m^−2^ underwent an initial screening session to assess eligibility. Height, weight, blood pressure, and body composition (by dual-energy X-ray absorptiometry; Discovery A, Hologic; (National Health and Nutrition Examination Survey—Body composition analysis (NHANES BCA) enabled) were determined. Participants were deemed healthy based on their responses to a medical questionnaire. The screening sessions and experimental trials were separated by at least 3 days.

### Study design

Participants were randomly assigned to ingest a 400 mL beverage containing either 30 g milk-derived protein concentrate (MILK), or 30 g pea-derived protein concentrate (PEA). After beverage ingestion, the bottle was rinsed with 150 mL of water, which was also ingested by the participants. Milk-derived protein concentrate (Refit MPC80) was obtained from FrieslandCampina (Wageningen, the Netherlands), and pea-derived protein concentrate (Nutralys S85F) was supplied by Kellogg (Battle Creek, MI, USA). Participants were allocated to a treatment according to a block randomization list performed using a computerized randomizer (http://www.randomization.com/). An independent researcher was responsible for random assignment (*n* = 12 per group) and preparation of the study treatment beverages, which were sequentially numbered according to subject number. The beverages were provided in identical, non-transparent protein shakers.

### Diet and physical activity

Participants refrained from sports and strenuous physical activities (e.g., lifting heavy weights), and alcohol consumption for 3 days prior to the experimental trial. In addition, all participants were instructed to complete a food and activity record for 3 days prior to the experimental trial (see Supplemental Table 1 for an overview of participants’ habitual food intake in the 3 days prior to the experimental trial). The evening before the trial, all participants consumed a standardized meal containing 2.8 MJ, with 20% energy provided as carbohydrate, 65% as fat, and 15% as protein, before 10:00 PM after which they remained fasted.

### Experimental protocol

The procedures applied in this trial have previously been described elsewhere [23]. At ~ 7:30 AM, participants arrived at the laboratory in an overnight post-absorptive state. A cannula was inserted into an antecubital vein for stable isotope amino acid infusion. A second cannula was inserted retrogradely into a dorsal hand vein on the contralateral arm for arterialized blood sampling. To obtain arterialized blood samples, the hand was placed in a hot box (60 °C) for 10 min prior to blood sample collection.

After taking a baseline blood sample (t = -180 min), the plasma phenylalanine pool was primed with a single dose of L-[ring-^13^C_6_]-phenylalanine (2.25 µmol∙kg^−1^). Thereafter, a continuous intravenous infusion of L-[ring-^13^C_6_]-phenylalanine (0.05 µmol∙kg^−1^∙min^−1^) was initiated (*t* = − 180 min) using a calibrated IVAC 598 pump (San Diego, CA, USA). Subsequently, arterialized blood samples were collected at *t* = − 90, − 60 and − 30 min. At *t* = 0 min, an arterialized blood sample was obtained as well as a muscle biopsy from the *M. vastus lateralis*. Immediately following the muscle biopsy, participants ingested a 400 mL beverage corresponding to their randomized treatment allocation, i.e., MILK (*n* = 12), or PEA (*n* = 12). To minimize dilution of the steady-state plasma L-[ring-^13^C_6_]-phenylalanine precursor pool, the phenylalanine content of the protein drink was enriched with 3.85% L-[ring-^13^C_6_]-phenylalanine. Arterialized blood samples were then collected at *t* = 15, 30, 45, 60, 90, 120, 150, 180, 210, 240, and 300 min after protein ingestion in the post-prandial period. Blood samples were collected into EDTA-containing tubes and centrifuged at 1200* g* for 10 min at 4 °C. Aliquots of plasma were frozen in liquid nitrogen and stored at − 80 °C. Second and third muscle biopsies from the *M. vastus lateralis* were collected at *t* = 120 and *t* = 300 min to determine post-prandial skeletal muscle protein synthesis rates over the 0–120, 120–300, and 0–300 min post-prandial periods. Muscle biopsy collection was alternated between legs and obtained with the use of a 5 mm Bergström needle [34], custom-adapted for manual suction. Samples were obtained from separate incisions from the middle region of the *M. vastus lateralis*, ~ 15 cm above the patella and ~ 3 cm below entry through the fascia. Local anesthetic (1% xylocaine with adrenaline 1:100,000) was applied to numb the skin and fascia. Muscle samples were freed from any visible non-muscle material, immediately frozen in liquid nitrogen, and stored at − 80 °C until further processing. When the experimental protocol was complete, cannulae were removed and participants were provided with food and monitored for ~ 30 min before leaving the laboratory. For a schematic representation of the infusion protocol, see Fig. 1.

### Protein powder analysis

Batch-specific nitrogen contents for milk- and pea-derived protein concentrates were provided by the manufacturers. The protein content of the milk-derived protein was determined as nitrogen content × 6.38, and the protein content of pea-derived protein was determined as nitrogen × 6.25 [35, 36]. Amino acid contents of the protein powders were determined by acid hydrolysis in triplicate, and subsequent analysis of the free amino acids using ultra-performance liquid chromatography–mass spectrometry (UPLC–MS; ACQUITY UPLC H-Class with QDa; Waters, Saint-Quentin, France), as previously described [23]. The amino acid composition of the protein powders are presented in Table 2.

### Plasma analysis

Plasma glucose and insulin concentrations were analyzed using commercially available kits (ref. no. A11A01667, Glucose HK CP, ABX Diagnostics, Montpellier, France; and ref. no. HI-14 K, Millipore, St. Louis, MO, respectively). Plasma amino acid concentrations were determined by UPLC–MS, as previously described [23].

Plasma L-[ring-^13^C_6_]-phenylalanine enrichments were determined by gas chromatography–mass spectrometry (GC–MS; Agilent 7890A GC/5975C MSD; Agilent Technologies), as previously described [23]. In short, the free amino acids from deproteinized plasma samples were purified using cation exchange resin columns (AG 50W-X8, mesh size: 100–200, ionic form: hydrogen (Bio-Rad Laboratories, Hercules, CA, USA)), and subsequently converted to their tert-butyl dimethylsilyl (TBDMS) derivative before analysis by GC–MS.

Basal (post-absorptive) muscle protein synthesis rates were assessed to confirm that protein ingestion increases muscle protein synthesis rates. The single biopsy approach was applied to assess post-absorptive muscle protein synthesis rates without the need to collect an additional muscle biopsy [37]. In short, plasma protein obtained prior to tracer infusion (*t* = − 180 min) was used to determine background L-[ring-^13^C_6_]-phenylalanine enrichments. For this purpose, the plasma sample was precipitated by adding perchloric acid. Subsequently, similarly as for the myofibrillar protein fraction, the denaturized plasma protein pellet was hydrolyzed, passed over a cation exchange resin column (AG 50W-X8, mesh size: 100–200, ionic form: hydrogen (Bio-Rad Laboratories, Hercules, CA, USA)), and the resulting amino acid samples were derivatized to their N(O,S)-ethoxycarbonyl-ethylesters before being measured by gas chromatography-combustion-isotope ratio mass spectrometry (GC-IRMS; Mat 253, Thermo Scientific, Bremen, Germany) using a DB5MS (30 m) column (Agilent technologies, Santa Clara, Ca, USA), as previously described [23].

### Muscle analysis

Muscle analysis for the determination of muscle protein-bound L-[ring-^13^C_6_]-phenylalanine enrichments has previously been explained in detail [23]. In short, a piece of wet muscle (~ 50–70 mg) was homogenized and a myofibrillar protein-enriched fraction was obtained by removal of the collagen enriched fraction. Subsequently, the amino acids from the resulting dried myofibrillar protein-enriched fractions were liberated by adding 2 mL of 6 M HCl and heating to 110 °C for 16 h, passed over a cation exchange resin column (AG 50W-X8, mesh size: 100–200, ionic form: hydrogen (Bio-Rad Laboratories, Hercules, CA, USA)), and derivatized to their N(O,S)-ethoxycarbonyl-ethylesters. The ratio of ^13^C/^12^C of myofibrillar protein-bound phenylalanine was determined using GC-IRMS.

### Calculations

The plasma free and muscle protein-bound L-[ring-^13^C_6_]-phenylalanine enrichments were used to calculate fractional myofibrillar protein synthesis rates (%∙h^−1^). This calculation was performed by the standard precursor-product equation [38]:

$$FSR=\left(\frac{\left({E}_{b2}-{E}_{b1}\right)}{\left({E}_{precursor}\bullet t\right)}\right)\bullet 100$$

where E_b_ is the increment in myofibrillar protein-bound L-[ring-^13^C_6_]-phenylalanine enrichment (mole% excess, MPE) during the tracer incorporation period, and t is the tracer incorporation time in h. Weighted mean plasma L-[ring-^13^C_6_]-phenylalanine enrichments were calculated by taking the measured enrichment between consecutive time points and correcting for the time between these sampling time points (E_precursor_). For calculation of post-prandial FSR, skeletal muscle biopsy samples at *t* = 0, 120 and 300 min were used. For the calculation of basal FSR, E_b2_ represented the protein-bound L-[ring-^13^C_6_]-phenylalanine enrichments in muscle at *t* = 0 min, and E_b1_ represented the protein-bound L-[ring-^13^C_6_]-phenylalanine enrichments in plasma protein at *t* = − 180 min.

Net incremental area under curve (iAUC) was determined for plasma amino acid concentrations during the 5 h post-prandial period following protein ingestion. The iAUC was calculated using the trapezoid rule, with plasma concentrations before beverage ingestion (*t* = 0 min) serving as baseline. Time to reach peak plasma amino acid concentrations were determined for each individual and subsequently averaged per group.

### Outcome measures

Myofibrillar FSR over the entire (i.e., 0 300 min) post-prandial period, comparing MILK *vs* PEA was defined as the primary outcome measure. Secondary outcome measures were myofibrillar FSR in the early (i.e., 0–120 min) and late (i.e., 120–300 min) post-prandial period, plasma glucose, insulin, and amino acid concentrations and plasma amino acid iAUC. Plasma glucose, insulin, and amino acid peak concentrations and time to peak were tertiary outcomes.

### Statistical analysis

A sample size calculation was performed with differences in post-prandial myofibrillar FSRs between the 2 treatments as primary outcome measure. Based on previous work in this area, a sample size of 12 participants per treatment, including a 10% dropout rate was calculated using a power of 80%, a significance level of 0.05, a difference in FSR of 0.008%∙h^−1^ (or ~ 20% when expressed as relative difference, e.g., 0.040 vs 0.048%∙h^−1^) [39], and a within-group standard deviation of 0.0065%∙h^−1^ (or ~ 16%) [40, 41].

The primary outcome, post-prandial (0–300 min) muscle protein synthesis rates between the two treatments, was analyzed by independent samples *t*-test. Likewise, basal post-absorptive, (− 180–0 min) and post-prandial myofibrillar protein synthesis rates during the early (0–120 min) and late (120–300 min) post-prandial period were analyzed by independent samples *t*-test. As secondary analyses, a two-way repeated measures ANOVA was performed to evaluate changes over time and the increase in post-prandial muscle protein synthesis rates above basal post-absorptive rates. Plasma glucose, insulin, and amino acid concentrations and amino acid enrichments over time were compared between groups using a two-way (*time x treatment*) repeated measures ANOVA, with time as within-subjects factor, and treatment as between-subjects factor. In case a significant *time x treatment* interaction was observed, post-hoc analyses were performed to determine significant differences between treatments for each time point. Participants’ characteristics, plasma glucose, insulin, and amino acid concentrations, expressed as peak values, time to peak and iAUC, were analyzed by independent samples *t*-test to locate differences between groups. Statistical analyses were performed with a software package (IBM SPSS statistics for Windows, version 26.0, IBM Corp., Armonk, NY, USA). Means were considered to be significantly different for *P* values < 0.05. Data are expressed as means ± SD. Except for plasma insulin concentrations (*n* = 11 for MILK), no missing values were present for any of the outcome parameters.

## Results

### Participants’ characteristics

Twenty-four healthy, recreationally active males (24 ± 3 years; 1.77 ± 0.06 m; 71.6 ± 8.9 kg) volunteered to participate in this parallel-group, double-blind, randomized controlled trial (Table 1).

**Table 1:** Participants’ characteristics

	MILK	PEA
Age (y)	26 ± 4	23 ± 2
Height (m)	1.76 ± 0.06	1.77 ± 0.07
Body mass (kg)	71.5 ± 9.0	71.7 ± 9.1
BMI (kg∙m^−2^)	23.0 ± 2.1	22.7 ± 1.9
Systolic blood pressure (mmHg)	119 ± 6	122 ± 12
Diastolic blood pressure (mmHg)	71 ± 9	69 ± 8
Resting heart rate (bpm)	64 ± 10	63 ± 8
Lean mass (kg)	53.2 ± 7.9	53.6 ± 6.8
Body fat (%)	23.1 ± 3.2	22.2 ± 4.0

Values represent mean ± standard deviation. n = 12 per nutritional intervention group. MILK: 30 g milk-derived protein, PEA: 30 g pea-derived protein. Independent samples *T*-test all *P* > 0.05

### Plasma glucose and insulin concentrations

**Fig. 2:**
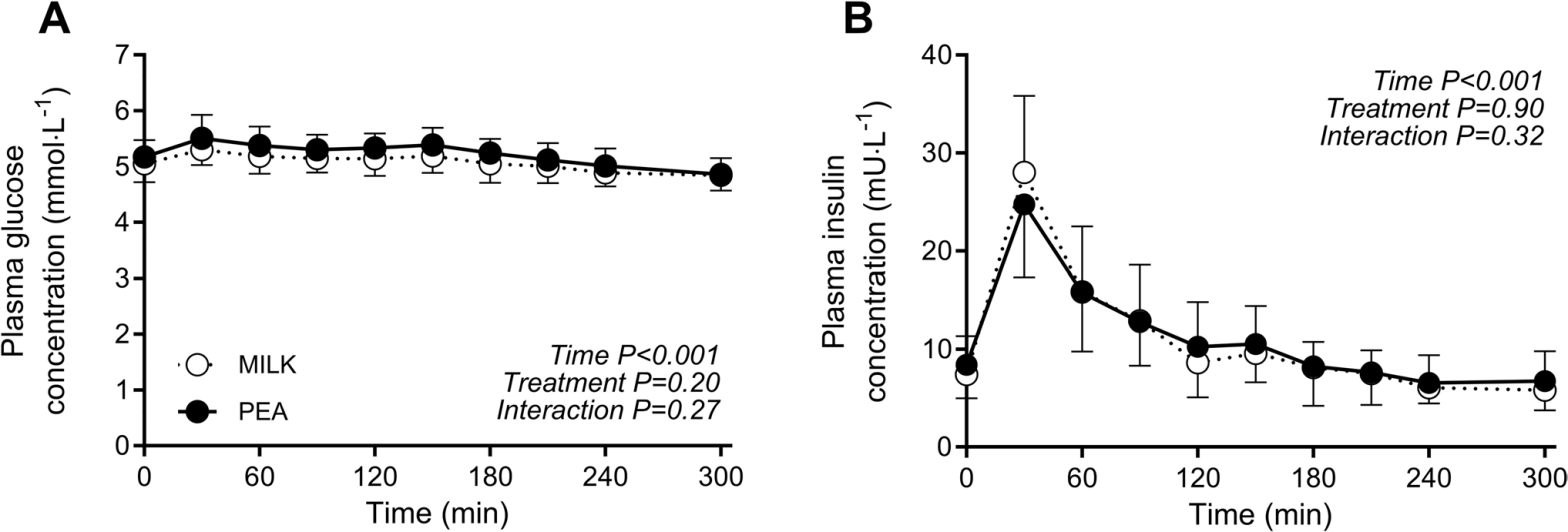
Post-prandial plasma glucose (Panel **A**) and insulin (Panel **B**) concentrations during the 5-h period following the ingestion of MILK *vs* PEA in healthy, young males (*n* = 12 per group). Time 0 min represents time of beverage intake. MILK: 30 g milk-derived protein, PEA: 30 g pea-derived protein. Values represent means ± standard deviation; repeated measures ANOVA with time as within-subjects variable and interventional drink (treatment) as between-subjects variable

Plasma glucose concentrations were maintained following protein ingestion, with no differences between treatments (*time x treatment*: *P* = 0.27; Fig. 2A). Plasma insulin concentrations increased following protein ingestion, with no differences between the MILK and PEA treatment group over time (*time x treatment*: *P* = 0.32; Fig. 2B). Similarly, peak plasma insulin concentrations (28 ± 8 *vs* 25 ± 7 mU∙L^−1^, respectively; independent samples *t*-test: *P* = 0.34), and post-prandial plasma insulin availability (iAUC) did not differ following MILK *vs* PEA ingestion (1058 ± 331 *vs* 797 ± 498 mU∙300 min∙L^−1^, respectively; independent samples *t*-test: *P* = 0.16).

### Plasma amino acid concentrations

**Fig. 3:**
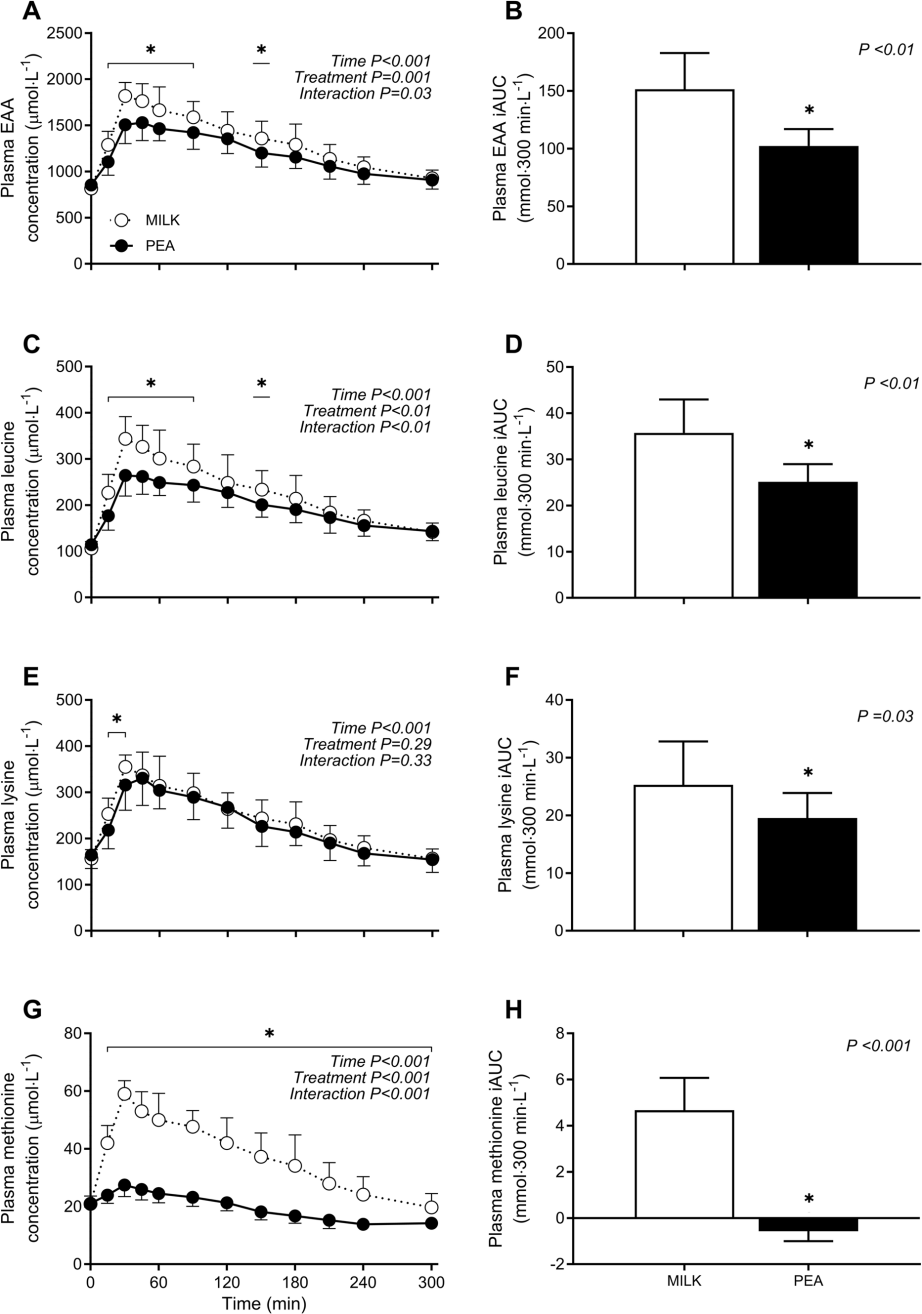
Post-prandial plasma essential amino acid (EAA, Panel **A**), leucine (Panel **C**), lysine (Panel **E**), and methionine (Panel **G**) concentrations during the 5 h post-prandial period following the ingestion of MILK *vs* PEA in healthy, young males (*n* = 12 per group). Time 0 min represents time of beverage intake. Panels **B**, **D**, **F** and **H** represent the 0–5 h incremental area under curve (iAUC) following protein ingestion. MILK: 30 g milk-derived protein, PEA: 30 g pea-derived protein. Values represent means ± standard deviation; *significantly different for MILK *vs* PEA (*P* < 0.05). Repeated measures ANOVA with time as within-subject variable and interventional drink (treatment) as between-subject variable

Plasma EAA concentrations increased following protein ingestion, with a greater rise in circulating EAA concentrations following MILK *vs* PEA ingestion (*time x treatment: P* = 0.03; Fig. 3A). Plasma EAA concentrations were increased above basal post-absorptive concentrations for the entire 300 min post-prandial period after MILK and PEA ingestion. In accordance with the significant *time x treatment* interaction, peak plasma EAA concentrations following MILK *vs* PEA ingestion were reached at 36 ± 10 min and 56 ± 32 min (independent samples *t*-test: *P* = 0.05), reaching levels of 1871 ± 124 and 1601 ± 162 µmol∙L^−1^ (independent samples *t*-test: *P* < 0.001), respectively. The overall increase in plasma EAA availability over the entire 300 min post-prandial period, expressed as iAUC, was ~ 48% greater for MILK *vs* PEA (151 ± 31 *vs* 102 ± 15 mmol∙300 min∙L^−1^; independent samples *t*-test: *P* < 0.001; Fig. 3B).

The post-prandial increase in plasma leucine concentrations following protein ingestion (Fig. 3C) differed between MILK *vs* PEA (*time x treatment: P* < 0.01). Plasma leucine concentrations increased for the entire 300 min post-prandial period following ingestion of both MILK and PEA. In accordance with the significant *time x treatment* interaction, peak plasma leucine concentrations were ~ 25% higher for MILK *vs* PEA (353 ± 45 *vs* 282 ± 30 µmol∙L^−1^, respectively; *P* < 0.001) and were reached 46 ± 43 and 58 ± 31 min after protein ingestion, respectively (independent samples *t*-test: *P* = 0.47). The overall increase in plasma leucine availability over the entire 300 min post-prandial period, expressed as iAUC, was ~ 44% greater for MILK *vs* PEA (36 ± 7 *vs* 25 ± 4 mmol∙300 min∙L^−1^; independent samples *t*-test: *P* < 0.001; Fig. 3D).

The post-prandial increase in plasma lysine concentrations following protein ingestion was not different following MILK *vs* PEA ingestion (*time x treatment P* = 0.33; Fig. 3E). Plasma lysine concentrations increased for 240 and 210 min after MILK and PEA ingestion, respectively. Peak plasma lysine concentrations were not different following MILK *vs* PEA ingestion (370 ± 29 *vs* 339 ± 50 µmol∙L^−1^, respectively; independent samples *t*-test: *P* = 0.08), but were reached ~ 16 min earlier (34 ± 7 vs 50 ± 21 min after protein ingestion respectively, independent samples *t*-test: *P* = 0.02). Peak plasma lysine concentrations increased ~ 137% above baseline values for MILK, and ~ 106% above baseline for PEA. Consequently, the overall increase in plasma lysine availability over the entire 300 min post-prandial period, expressed as iAUC, was ~ 25% greater for MILK *vs* PEA (25 ± 8 *vs* 20 ± 4 mmol∙300 min∙L^−1^; independent samples *t*-test: *P* = 0.03; Fig. 3F).

The post-prandial increase in plasma methionine concentrations following protein ingestion was significantly greater following MILK *vs* PEA ingestion (*time x treatment: P* < 0.001; Fig. 3G). Plasma methionine concentrations increased for 240 and 90 min after MILK and PEA ingestion, respectively. After which methionine concentrations became lower when compared to post-absorptive values in the PEA group. In accordance with the significant *time x treatment* interaction, peak plasma methionine concentrations were ~ 114% greater for MILK *vs* PEA (60 ± 5 and 28 ± 4 µmol∙L^−1^, independent samples *t*-test: *P* < 0.001), and reached ~ 30 min after protein ingestion (34 ± 9 *vs* 35 ± 22 min; independent samples *t*-test: *P* = 0.86). As a result, peak plasma methionine concentrations increased ~ 190% above baseline values for MILK, but only increased ~ 33% above baseline values for PEA. The overall increase in plasma methionine availability over the entire 300 min post-prandial period, expressed as iAUC, was several fold greater for MILK *vs* PEA (4.7 ± 1.4 *vs* − 0.6 ± 0.4 mmol∙300 min∙L^−1^; independent samples *t*-test: *P* < 0.001; Fig. 3H).

In general, post-prandial increases in plasma amino acid concentrations revealed significant differences over time following MILK *vs* PEA ingestion for most amino acids (Supplemental Fig. 2; *time x treatment: P* < 0.05). The post-prandial increases in plasma alanine, BCAA, cystine, proline, threonine, tryptophan, tyrosine, and valine availability over the entire 300 min post-prandial period (iAUC) were greater for MILK *vs* PEA (independent samples *t*-test: *P* < 0.05), with an exception for plasma arginine, asparagine, glycine, and ornithine, which were lower for MILK *vs* PEA (independent samples *t*-test: *P* < 0.05, Supplemental Fig. 2).

### Plasma free and muscle tissue L-[ring-^13^C_6_]-phenylalanine enrichments

**Fig. 4:**
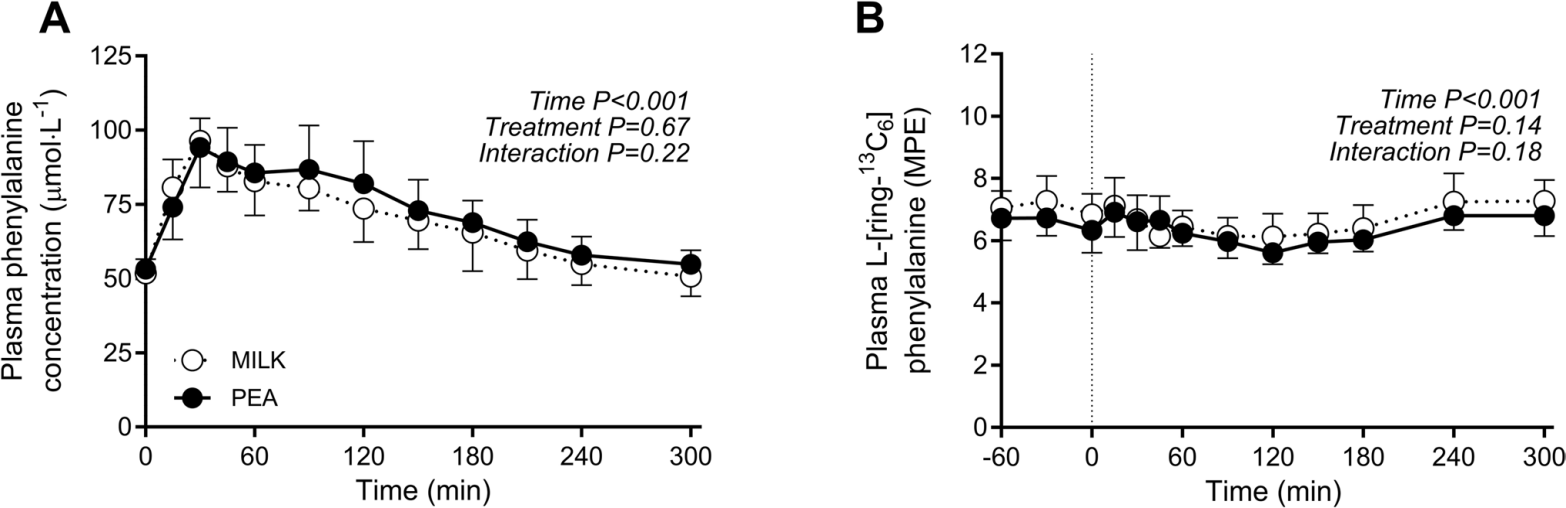
Post-prandial plasma phenylalanine concentrations (Panel **A**) and plasma L-[ring-^13^C_6_]-phenylalanine enrichments (Panel **B**) during the 5 h post-prandial period following the ingestion of MILK *vs* PEA in healthy, young males (*n* = 12 per group). Time 0 min represents time of beverage intake. MILK: 30 g milk-derived protein, PEA: 30 g pea-derived protein. Values represent means ± standard deviation. Repeated measures ANOVA with time as within-subject variable and interventional drink (treatment) as between-subject variable

Plasma L-phenylalanine concentrations and L-[ring-^13^C_6_]-phenylalanine enrichments over time are presented in Fig. 4A and 4B, respectively. Plasma L-[ring-^13^C_6_]-phenylalanine enrichments over time did not differ following MILK *vs* PEA ingestion during the post-prandial period (*time x treatment: P* = 0.18). Mean plasma L-[ring-^13^C_6_]-phenylalanine enrichments averaged 7.11 ± 0.65 and 6.63 ± 0.58 MPE during the basal post-absorptive period (independent samples *t*-test: *P* = 0.07), and 6.64 ± 0.53 and 6.33 ± 0.27 MPE throughout the 5 h post-prandial period (independent samples *t*-test: *P* = 0.08) following MILK and PEA ingestion, respectively. Myofibrillar protein-bound L-[ring-^13^C_6_]-phenylalanine enrichments were higher following ingestion of MILK and PEA from 0.0032 ± 0.0032 and 0.0028 ± 0.0029 MPE at *t* = 0 min, to 0.0115 ± 0.0041 and 0.0104 ± 0.0035 MPE at t = 120 min, reaching 0.0214 ± 0.0049 and 0.0205 ± 0.0047 MPE at t = 300 min after protein ingestion, respectively.

### Muscle protein synthesis rates

**Fig. 5:**
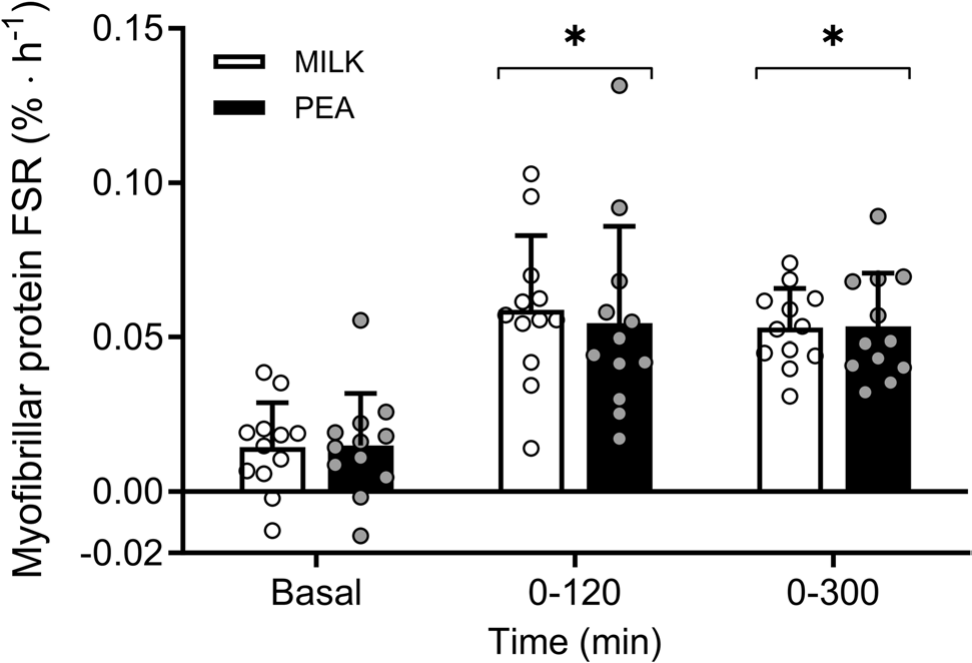
Myofibrillar protein fractional synthetic rates (FSR) at different time points following ingestion of MILK *vs* PEA in healthy, young males (*n* = 12 per group). MILK: 30 g milk-derived protein, PEA: 30 g pea-derived protein. Bars represent means ± standard deviation, dots represent individual values. *significantly effect of time *P* < 0.001

Post-absorptive fractional myofibrillar protein synthesis rates averaged 0.014 ± 0.014 and 0.015 ± 0.017%∙h^−1^ in the MILK and PEA experiment, with no differences between the groups (independent samples *t*-test: *P* = 0.94). The primary outcome, post-prandial muscle protein synthesis rates (0–300 min), did not differ between MILK *vs* PEA, (0.053 ± 0.013 *vs* 0.053 ± 0.017%∙h^−1^, independent samples *t*-test: *P* = 0.96, Fig. 5), neither did the increase above basal post-absorptive rates (change scores, independent samples *t*-test: *P* = 0.99). In addition, muscle protein synthesis rates did not differ for the early (0–120 min; independent samples *t*-test: *P* = 0.71), and late (120–300 min; independent samples *t*-test: *P* = 0.55) post-prandial period. Secondary analyses using two-way repeated measure ANOVA showed that protein ingestion increased myofibrillar protein synthesis rates to 0.059 ± 0.024 and 0.054 ± 0.031%∙h^−1^ during the early post-prandial period (0–120 min) and to 0.049 ± 0.017 and 0.053 ± 0.015%∙h^−1^ during the late post-prandial period (120–300 min) in MILK and PEA, respectively (main effect of time *P* < 0.001), with no *time x treatment* interaction.

## Discussion

The present study shows that ingestion of a pea-derived protein is followed by a substantial increase in muscle protein synthesis rates in healthy, young males. Despite lower post-prandial plasma essential amino acid concentrations, post-prandial muscle protein synthesis rates following the ingestion of 30 g pea-derived protein did not differ from the rates observed after ingesting an equivalent amount of milk-derived protein.

Plant-derived proteins are known to have deficiencies in specific EAA according to the WHO/FAO/UNU requirements [32], and can be particularly low in leucine, lysine, and/or methionine contents [26]. Pea-derived protein contains a sufficient amount of leucine and a lysine content that is higher than most plant-derived protein sources [26]. In contrast, pea-derived protein has a particularly low methionine content [26]. In the present study, EAA (9.8 *vs* 7.7 g), leucine (2.4 *vs* 1.8 g), and methionine (0.7 *vs* 0.2 g) contents were all substantially higher in the milk compared with the pea-derived protein (Table 2). Furthermore, although pea-derived protein is considered to be very rich in lysine, its content was still lower when compared to milk-derived protein (Table 2). These differences in amino acid composition translated into lower post-prandial peak plasma EAA, leucine, and methionine concentrations (Fig. 3) and a lesser post-prandial plasma amino acid availability (Fig. 3) following ingestion of a single bolus of 30 g pea- when compared with milk-derived protein. The observed differences in post-prandial plasma amino acid profiles appear to be in line with previous publications showing an attenuated rise in circulating plasma amino acids following ingestion of various plant-derived proteins (such as soy, wheat, and potato protein) when compared with the ingestion of an equivalent amount of animal-derived protein [22–24]. The attenuated amino acid response may be attributed to differences in protein structure and function of plant-derived proteins that may compromise digestion and amino acid absorption and/or amino acid retention in splanchnic tissues [2, 42–44]. In this study we assessed whether such differences in the post-prandial amino acid responses also lead to differences in post-prandial muscle protein synthesis rates.

**Table 2:** Amino acid composition of proteins consumed

	MILK	PEA
Alanine	0.9	1.1
Arginine	0.8	1.7
Aspartic acid	1.8	2.5
Cystine	0.1	0.1
Glutamic acid	5.1	3.9
Glycine	0.5	1.1
Histidine	0.6	0.5
Isoleucine	0.9	0.6
Leucine	2.4	1.8
Lysine	2.0	1.7
Methionine	0.7	0.2
Phenylalanine	1.2	1.2
Proline	2.9	1.1
Serine	1.2	1.4
Threonine	0.9	0.8
Tyrosine	0.6	0.4
Valine	1.1	0.8
TAA	23.8	20.9
EAA	9.8	7.7
BCAA	4.4	3.2
Nitrogen content (%)	13.4	13.6
Protein content (%)	85.5^1^	84.7^2^

Values for amino acid contents are in grams per 30 g protein. ^1^Protein as nitrogen content * 6.38; ^2^Protein as nitrogen content *6.25; MILK: 30 g milk-derived protein, *PEA* 30 g pea-derived protein. *BCAA* branched chain amino acids, *EAA* essential amino acids, *TAA* total amino acids

The post-prandial rise in plasma amino acid concentrations following the ingestion of pea-derived protein resulted in a strong post-prandial stimulation of muscle protein synthesis (Fig. 5). Interestingly, we show that despite the lower post-prandial plasma amino acid availability following pea- *vs* milk-derived protein ingestion, the post-prandial muscle protein synthetic response to pea-derived protein did not differ from milk-derived protein ingestion. Clearly, the provided pea-derived protein has sufficient potential to strongly stimulate muscle protein synthesis in vivo in humans. This is in line with our previous work [19], demonstrating that the ingestion of sufficient amounts (e.g., 30 g) of wheat- or potato-derived protein does not result in a lesser muscle protein synthetic response when compared to the ingestion of an equivalent amount of dairy protein in young individuals, despite a low(er) lysine and/or methionine availability. Consequently, we need to conclude that overall plasma amino acid availability, as a resultant of both endogenous and exogenous amino acid release, is sufficient to allow maximal stimulation of post-prandial muscle protein synthesis rates following the ingestion of pea-derived protein. Collectively, these findings imply that pea-derived protein represents a viable, high-quality protein source to support human nutrition, and further research might consider its utility in a wider range of contexts.

To date, most studies comparing anabolic properties of animal- versus non-animal proteins have assessed muscle protein synthesis rates following the ingestion of protein isolates or protein concentrates [14, 19–22, 24, 25]. However, our daily protein intake is generally not consumed in the form of protein isolates or concentrates, but rather in the form of whole-foods. The matrix in which proteins are embedded in whole-foods can differ substantially between animal- and non-animal protein sources [45–47]. Most plant-based whole-foods contain anti-nutritional factors (e.g., dietary fiber, trypsin inhibitors or phytates) that compromise protein digestibility, attenuate the post-prandial rise in circulating amino acid concentrations and, as such, lower the capacity to increase muscle protein synthesis rates [48, 49]. Therefore, our data are restricted to (pea and milk) protein concentrates and are not necessarily reflective of the metabolic response to the ingestion of all (pea and milk) derived products. Furthermore, it should be noted that a specific amino acid deficiency of a protein or protein source may be compensated for by other proteins or protein sources, as most proteins or protein sources are typically consumed as part of a more complex meal or protein blend [47]. Therefore, we would encourage the exploration of anabolic responses to the ingestion of protein sources in the form of whole-foods and more complex, composite meals. The latter may provide even more insight in the impact of our food processing and consumption on post-prandial protein handling and subsequent muscle maintenance.

In conclusion, ingestion of 30 g pea-derived protein stimulates muscle protein synthesis rates in young, healthy males. Post-prandial muscle protein synthesis rates following the ingestion of 30 g pea-derived protein do not differ from rates observed after ingesting 30 g milk-derived protein. Ingestion of a meal-like (30 g) dose of plant-derived protein can be as effective as ingesting an equivalent amount of animal-derived protein to increase muscle protein synthesis rates in vivo in healthy, young males.

### Supplementary Information

Below is the link to the electronic supplementary material.

Supplementary file1 (DOCX 11347 KB)

## Data Availability

Data described in the manuscript will be made available upon request.

## Abbreviations

AA: Amino acid
ANOVA: Analysis of variance
BCAA: Branched-chain amino acids
BMI: Body mass index
CTCM: Clinical Trial Centre Maastricht
DEXA: Dual-energy X-ray absorptiometry
EAA: Essential amino acid
FSR: Fractional synthetic rate
GC-IRMS: Gas chromatography-combustion-isotope ratio mass spectrometry
GC–MS: Gas chromatography–mass spectrometry
MILK: 30 G milk-derived protein concentrate
MPE: Mole percent excess
MPS: Muscle protein synthesis
MyoPS: Myofibrillar protein synthesis
NEAA: Non-essential amino acid
NHANES BCA: National Health and Nutrition Examination Survey—Body composition analysis
PEA: 30 G pea-derived protein concentrate
TAA: Total amino acid
UPLC–MS: Ultra-performance liquid chromatography–mass spectrometry
